# The Therapeutic Effect of Exogenous Melatonin on Depressive Symptoms: A Systematic Review and Meta-Analysis

**DOI:** 10.3389/fpsyt.2022.737972

**Published:** 2022-03-17

**Authors:** Cancan Li, Dandan Ma, Min Li, Tao Wei, Xuan Zhao, Yuanyuan Heng, Delong Ma, Enoch Odame Anto, Yanbo Zhang, Mingyun Niu, Wangxin Zhang

**Affiliations:** ^1^School of Public Health, Shandong First Medical University and Shandong Academy of Medical Sciences, Taian, China; ^2^Beijing Key Laboratory of Clinical Epidemiology, School of Public Health, Capital Medical University, Beijing, China; ^3^The Second Affiliated Hospital of Shandong First Medical University, Taian, China; ^4^School of Basic Medical Science, Shandong First Medical University and Shandong Academy of Medical Sciences, Taian, China; ^5^Department of Medical Image, Shandong Provincial Hospital, Cheeloo College of Medicine, Shandong University, Jinan, China; ^6^Department of Medical Image, Taian City Central Hospital, Taian, China; ^7^College of Health Sciences, Department of Medical Diagnostic, Kwame Nkrumah University of Science and Technology, Kumasi, Ghana; ^8^Centre for Precision Health, School of Medical and Health Sciences, Edith Cowan University, Perth, WA, Australia

**Keywords:** melatonin, depression, depressive symptoms, therapeutic effect, systematic review

## Abstract

**Background:**

Depression-related mortality and morbidity pose growing public health burdens worldwide. Although the therapeutic effect of exogenous melatonin on depression has been investigated, findings remain inconsistent. We conducted this systematic review and meta-analysis to clarify the effectiveness of melatonin in the treatment of depression, including primary and secondary depression symptoms.

**Methods:**

We searched the online databases of PubMed, EMBASE, and the Cochrane Library for original studies published up to May 2021. We used STATA 14.0 software to synthesize the results of included studies. To evaluate the effectiveness of melatonin, we calculated the standardized mean differences (SMDs) and 95% confidence intervals (CIs) of depression scores between the melatonin and placebo groups.

**Results:**

Our literature search returned 754 publications, among which 19 studies with 1,178 patients (715 women, 463 men; mean age: 56.77 years) met inclusion criteria. Melatonin dosages ranged from 2 to 25 mg per day; treatment durations were between 10 days and 3.5 years. Our synthesized results showed that melatonin was not found significantly beneficial for alleviating depressive symptoms (SMD = −0.17, 95% CI = [−0.38, 0.05]). Subgroup analysis demonstrated that the decrease in depression scores measured with the Beck Depression Inventory (BDI) was significant (SMD = −0.52, 95% CI = [−0.73, −0.31]).

**Conclusions:**

There is very limited evidence for effects of melatonin on depression.

## Introduction

Depression, one of the most common psychiatric disorders, contributes to morbidity and mortality in adults and adolescents worldwide (1). The World Health Organization reported that the number of people suffering from depression increased by more than 18% from 2005 to 2015 (2). Depression is considered as a heterogeneous disease on account of complex pathological mechanisms and multiple etiologies (1). Currently, the efficacy for depression therapy is limited due to the need for long-term treatment and variation in symptom presentation (2). Antidepressant medication lacks uniform effectiveness. The large treatment-refractory population indicates the need to explore additional therapeutic agents, such as novel antidepressive treatments that have fewer side effects (3, 4). Nevertheless, successful treatment is believed to enable a dramatic improvement in patients' overall functioning and quality of life (5).

Melatonin, also known as 5-methoxy-N-acetyltryptamine, is a pleiotropic neurohormone secreted by the pineal gland at low levels during the daytime and high levels at night (6). Disorders of melatonin secretion were found to be historically associated with depression; however, when used as a therapy for this condition, exogenous melatonin is thought to function by normalizing patients' disrupted circadian architecture *via* binding to two receptors (MT_1_ and MT_2_) in the suprachiasmatic nucleus and other parts of the brain (7–10).

At present, studies exploring the therapeutic impact of exogenous melatonin on depression treatment have shown inconsistent results (11–16). In 2014, a systematic review and meta-analysis revealed that exogenous melatonin had no therapeutic or prophylactic effect on depression (17). Another systematic review involving three trials reported a non-significant effect of melatonin on treating depressive episodes (18). However, five newly published randomized controlled trials (RCTs) demonstrated the beneficial effects of melatonin in depression treatment (14, 19–22). In these five studies, melatonin was treated for 12 weeks, and the participants were with moderate to severe depression. While the previously published studies that did not testify the effectiveness of melatonin involved participants with mild depression symptom. Here, we report a systematic review and meta-analysis and provide updated findings on the effectiveness of melatonin on depression treatment including primary and secondary depression symptoms.

## Methods

Our study was conducted in accordance with the Preferred Reporting Items for Systematic Reviews and Meta-Analyses (Supplementary Table S1 and Supplementary Box 1).

### Inclusion and Exclusion Criteria

Original studies that met the following criteria were included in our meta-analysis: (1) study participants were adults (aged ≥ 18 years); (2) the effect of melatonin on remission of depression was investigated among participants with depressive symptoms; (3) RCTs or randomized crossover trials were performed; (4) the study involved the diagnosis and/or measurement of depression; (5) the severity of depression was rated by a self- or clinician-administered questionnaire; (6) the mean severity of depressive symptoms was compared between melatonin and placebo groups; (7) the dose and mode of administration were reported; and (8) the study was published in English.

Exclusion criteria were as follows: (1) melatonin was not given as an intervention; (2) no scores on depressive symptoms were reported in the melatonin and placebo groups; and (3) the effect of melatonin or placebo was not reported separately from other interventions.

### Search Strategies and Study Screening

We searched PubMed, EMBASE, and the Cochrane Library for trials published up to May 15, 2021.

The terms “melatonin, depression, depressive disorders, mood disorders, depressive symptoms, treat^*^, effect^*^, and therapeutics” were used for the literature search (17).

To improve the search strategy, we used restriction options in accordance with the population, intervention, comparison, and outcomes principle: study participants were selected from an adult population; the intervention agent was melatonin; the comparison agent was placebo; and the outcome was the relevant score for depressive symptoms as measured by investigators. Specific search parameters were as follows: (1) PubMed: clinical trial, RCT, humans, adults, English language; (2) EMBASE: controlled clinical trial, RCT, clinical study, humans, 18+ years, English language; (3) the Cochrane Library: no restrictions.

Two authors (CL and DaM) independently reviewed the literature to screen original studies by reading the title, abstract, and/or full text of each. References cited in the included studies were also screened for eligibility. All inconsistencies and disagreements were resolved through discussion with a third author (ML).

### Data Collection

Two authors (CL and DaM) extracted data from the selected studies, such as the study characteristics, intervention, primary outcomes, measurement instruments, and adverse events. For studies that provided the mean and standard deviation (SD) of depression scores, we recorded summary data directly; otherwise, we calculated summary data using statistical approaches. Of studies in which the 95% confidence interval (CI) was reported, the SD was calculated using the following equation:


$$\begin{array}{l} \text{SD}=\sqrt{n}\times \left(\text{upper limit}-\text{lower limit}\right)/3.92 \end{array}$$

In studies in which the median and interquartile range (IQR) were reported, the mean and SD were computed as follows (23):


$$\begin{array}{l} \text{ mean}\approx \text{median}\\ \text{SD}\approx \text{Norm IQR }=\;\left(P_{75}-P_{25}\right)\times 0.7413 \end{array}$$

### Statistical Analysis

The meta-analysis was conducted with STATA 14.0 software (Stata Corp, College Station, TX, USA). The standardized mean difference (SMD) and 95% CI were synthesized to evaluate the difference in depression scores between the melatonin and placebo groups. Statistical heterogeneity was examined on the basis of Cochran's Q test and the *I*^2^ statistic. When *p* <0.10 and *I*^2^ > 50%, original studies were regarded as demonstrating heterogeneity, and a random-effect model meta-analysis was undertaken; otherwise, a fixed-effect model meta-analysis was carried out. Subgroup analyses were also performed on the basis of depression scales, melatonin dosage, and/or treatment duration. To assess the robustness of the meta-analysis, sensitivity analysis was conducted by removing each original study one at a time. Publication bias was identified through funnel plot analysis along with Egger's regression asymmetry test. In terms of the quality of each study, the risk of bias was assessed *via* Review Manager Version 5.4 (Cochrane Collaboration, Oxford, UK), including seven domains with each domain containing three types of risk (23).

## Results

### Literature Search

**Figure 1:**
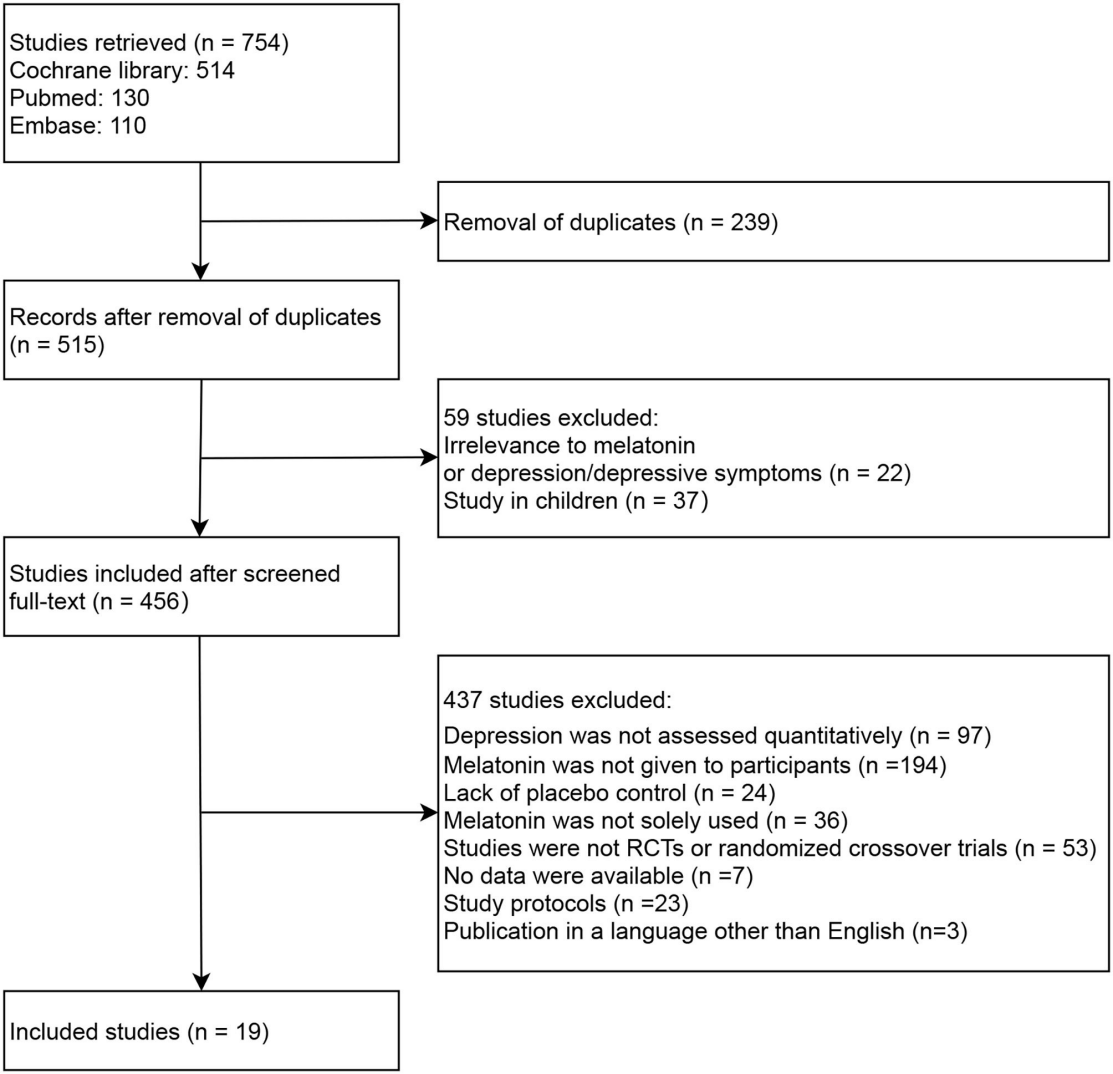
Flow diagram of literature screening.

The literature search and screening process is depicted in Figure 1. Our initial search returned 754 records, among which 239 studies were removed due to being duplicates between databases. Upon reviewing each source's title and/or abstract, 59 studies were excluded for the following reasons: (1) irrelevance to melatonin or depression/depressive symptoms (*n* = 22); or (2) study in children (*n* = 37). Of the 456 full-text studies first reviewed, 437 were excluded for the following reasons: (1) depression was not assessed quantitatively (*n* = 97); (2) melatonin was not given to participants (*n* = 194); (3) lack of placebo control (*n* = 24); (4) melatonin was not solely used (*n* = 36); (5) studies were not RCTs or randomized crossover trials (*n* = 53); (6) no data were available (*n* = 7); (7) study protocols (*n* = 23); (8) publications in a language other than English (*n* = 3). Consequently, 19 studies were included in the meta-analysis, among which 15 were RCTs (14–16, 19–22, 24–31) and four were randomized crossover trials (32–35).

### Characteristics of Included Studies

As shown in Table 1, a total of 1,178 participants (715 women, 463 men; mean age: 56.77 years) were involved in the original studies, ranging from 17 to 237 patients per trial. The duration of melatonin treatment was from 10 days to 3.5 years, and the melatonin dosage ranged from 2 to 25 mg per day. Two melatonin formulations are available: slow- or fast-release. Individuals in three studies were administered slow-release melatonin (15, 30, 33). Most subjects suffered secondary depression syndrome stemming from other psychiatric or physical disorders rather than primary depression. With regard to depression measurement, eight scales were employed to quantify the severity of depressive symptoms: the Beck Depression Inventory (BDI); Hospital Anxiety and Depression Scale (HADS-D); Center for Epidemiologic Studies Depression (CES-D); Major Depression Inventory (MDI); 21-item Hamilton Depression Rating Scale (HDRS-21); Yesavage Geriatric Depression Scale (GDS); Cornell Scale for Depression in Dementia (CSDD); and the 8-item addendum of the Structured Interview Guide for the Hamilton Depression Rating Scale–Seasonal Affective Disorders Version Self-Rating Format (ATYP).

**Table 1:** Characteristics of the included studies.

**Author**	**Country**	**N. participants (N. loss to follow-up)**	**Age**	**Scales of depression**	**Basic medicine/other treatment**	**Comorbidities**	**Primary depression**	**Melatonin and placebo administration**	**Treatment duration and depression score after treatment**	**Adverse events**
Chojnacki et al. (24)	Poland	64 (4)	MEL: 35.6 ± 11.4	BDI	Mesalazine	Ulcerative colitis	No	5 mg melatonin or placebo daily at bedtime for 3 months	12 weeks: 16.7 ± 3.5 (MEL)	Headaches
		F: 38, M: 22	PLC: 33.9 ± 11.7						12 weeks: 17.7 ± 2.5 (PLC)	
Ghaderi et al. (19)	Iran	60 (6)	MEL: 42.5 ± 8.0	BDI	Methadone maintenance treatment (MMT)	MMT patients	No	10 mg melatonin or placebo once a day, 1 hour before bedtime for 12 weeks	12 weeks: 19.5 ± 9.1 (MEL)	Headache, daytime sleepiness, and dizziness
		M: 60 (6)	PLC: 42.7 ± 9.9						12 weeks: 23.3 ± 8.8 (PLC)	
Daneshvar Kakhaki et al. (21)	Iran	60 (9)	MEL: 64.4 ± 8.2	BDI	Not available	Parkinson's disease	No	10 mg melatonin or placebo once a day, 1 h before bedtime for 12 weeks	12 weeks: 18.7 ± 4.1 (MEL)	Headache and daytime sleepiness
		F: 19, M:32	PLC: 66.3 ± 9.3						12 weeks: 21.4 ± 4.9 (PLC)	
Shabani et al. (20)	Iran	60 (2)	MEL: 26.5 ± 3.5	BDI	Not available	Polycystic ovary syndrome	No	10 mg melatonin or placebo once a day, 1 h before bedtime for 12 weeks	12 weeks: 16.9 ± 3.6 (MEL)	Not available
		F: 60 (2)	PLC: 26.0 ± 3.3						12 weeks: 20.6 ± 5.1 (PLC)	
Ostadmohammadi et al. (22)	Finland	60 (7)	MEL: 65.6 ± 13.1	BDI	None	Diabetic Hemodialysis	No	5 mg melatonin or placebo twice a day, 1 hour before bedtime for 12 weeks	12 weeks: 21.9 ± 3.9 (MEL)	None
		F: 15, M: 38	PLC: 64.1 ± 8.2						12 weeks: 24.8 ± 5.0 (PLC)	
Serfaty et al. (15)	UK	33 (2)	MEL: 38.1 ± 11.6	BDI and HDRS-21	Treatment as usual	Major depressive disorder	Yes	6 mg melatonin or placebo once a day at bedtime over 4 weeks	BDI: 5 weeks: 18.5 ± 11.6 (MEL)	Poor sleep, vivid dreams, day time sleepiness and fuzzy feeling
		F: 27, M: 4	PLC: 42.0 ± 12.6						5 weeks: 21.7 ± 9.6 (PLC)	
									HDRS-21 items: 4 weeks: 13.3 ± 6.7 (MEL)	
									4 weeks: 14.7 ± 5.8 (PLC)	
Palmer et al. (14)	Brazil	36 (0)	MEL: 54.24 ± 10.59	BDI	Adjuvant chemotherapy for breast cancer	Breast cancer	No	20 mg melatonin or placebo once a day, 1 h before bedtime	10 days: 6.71 ± 4.57 (MEL)	None
		F: 36 (0)	PLC: 54.11 ± 9.15						10 days: 14.56 ± 7.76 (PLC)	
Roostaei et al. (25)	Iran	26 (1)	MEL: 33.3 ± 7.6	BDI	Once weekly interferon beta	Multiple sclerosis	No	3 mg melatonin or placebo once a day for 12 months	12 months: 19.12 ± 8.6 (MEL)	None
		F: 21, M: 4	PLC: 34.5 ± 8.2						12 months: 14.3 ± 7.8 (MEL)	
Chen et al. (26)	USA	95 (9)	MEL: Mean 59 (range 48–80)	CES-D	Not available	Postmenopausal breast cancer	No	3 mg melatonin or placebo once a day for 4 months	12 weeks: 6.5 ± 4.6 (MEL)	None
		F: 95 (9)	PLC: Mean 59 (range 38–71)						12 weeks: 6.0 ± 5.4 (PLC)	
Peles et al. (32)	Israel	80 (19)	42.6 ± 1.2	CES-D	Methadone	Sleep disturbances	No	5 mg melatonin or placebo once a day for 6 weeks one arm, 1-week washout, 6 weeks other arm	6 weeks: 1.3 ± 0.1 (MEL)	Not available
		F: 24, M: 56							6 weeks: 1.2 ± 0.1 (PLC)	
Grima et al. (33)	Australia	33 (1)	37 ± 11	HADS-D	None	Traumatic brain injury	No	2 mg melatonin or placebo once a day before bedtime for 4 weeks one arm, 48-hour washout period, 4 weeks other arm	4 weeks: 8.53 (95% CI: 6.93–10.13) (MEL)	None
		F: 11, M: 22							4 weeks: 8.34 (95% CI: 6.75–9.94) (PLC)	
Lu et al. (34)	Singapore	24 (7)	Mean 41.2	HADS-D	None	Irritable bowel syndrome	No	3 mg melatonin or placebo once a day for 8 weeks one arm, 4-week washout period, 8 weeks other arm	8 weeks: 5.4 ± 2.6 (MEL)	Daytime sleepiness
		F: 24 (7)							8 weeks: 5.3 ± 2.8 (PLC)	
Song et al. (27)	Singapore	42 (2)	MEL: Mean 27.15 (SE 1.95)	HADS-D	None	Irritable bowel syndrome with sleep disturbances	No	3 mg melatonin or placebo daily at bedtime for 2 weeks	2 weeks: 4.80 ± 4.07 (MEL)	Not available
		F:24, M: 16	PLC: Mean 27.70 (SE 2.45)						2 weeks: 2.85 ± 2.15 (PLC)	
Madsen et al. (16)	Denmark	252 (15)	MEL: 62.90 ± 11.32	HADS-D and MDI	Not available	Acute coronary syndrome	No	25 mg melatonin or placebo daily 1 h before bedtime for 12 weeks	HADS-D 12 weeks: 1.19 (95% CI: 0.80–1.57) (MEL)	Altered bowel habits, Tachycardia, Dizziness
		F: 56, M: 196	PLC: 62.10 ± 10.81						12 weeks: 1.48 (95% CI: 1.09–1.88) (PLC)	
									MDI	
									2 weeks: 5.95 (95% CI: 5.02–6.87) (MEL)	
									2 weeks: 5.10 (95% CI: 4.30–5.80) (PLC)	
Hansen et al. (28)	USA	54 (8)	MEL: Mean 51 (IQR 46–66)	MDI	After surgery	Breast cancer	No	6 mg melatonin or placebo once a day for 3 months (2 weeks were included)	2 weeks: 6 (4–12) (IQR) (MEL)	Dizziness, headache, and paresthesia in the mouth region, arms, or legs
		F: 54 (8)	PLC: Mean 60 (IQR 49–68)						2 weeks: 10 (5.5–19.75) (IQR) (PLC)	
Garzon et al. (35)	Spain	22 (4)	F: Mean 74.3	GDS	Hypnotics (*n* = 14)	Sleep disorder	No	5 mg melatonin or placebo daily at bedtime for 8 weeks, 2 weeks washout, then crossover 8 weeks	8 weeks: 5.61 ± 1.15 (MEL)	None
		F: 15, M: 7	M: Mean 75.8						8 weeks: 7.06 ± 1.16 (PLC)	
Morales-Delgado et al. (31)	Mexico	40 (9)	MEL: 82.2 ± 5.8	GDS	None	Mild–moderate dementia	No	5 mg melatonin or placebo every night for 8 weeks	8 weeks: 4.9 ± 3.6 (MEL)	no serious adverse events
		F: 24, M: 7	PLC: 83.1 ± 7.4						8 weeks: 4 ± 1.7 (PLC)	
Riemersma-van der Lek et al. (29)	Netherlands	189 (0)	85.8 ± 5.5	CSDD	Bright light	Dementia	No	2.5 mg melatonin or placebo daily 1 h before bedtime for maximum 3.5 years	6 weeks: 7.5 ± 6.2 (MEL)	None
		F: 170, M: 19							6 weeks: 7.8 ± 5.2 (PLC)	
Leppämäki et al. (30)	Finland	58 (5)	MEL: 36.0 ± 9.5	ATYP	None	Subsyndromal seasonal affective disorder and/or weather-associated syndrome	Yes	2 mg melatonin or placebo daily 1–2 h before a desired bedtime for 3 weeks	3 weeks: 1.8 ± 2.8 (MEL)	Headaches
		F: 44, M: 14	PLC: 42.6 ± 10.8						3 weeks: 1.2 ± 2.6 (PLC)	

*CI, confidence interval; MEL, melatonin; PLC, placebo; M, male; F, female; IQR, interquartile range; SE, standard error; BDI, Beck Depression Inventory; HDRS-21, 21-item Hamilton Depression Rating Scale; CES-D, Center for Epidemiological Studies Depression Scale; HADS-D, the Hospital Anxiety and Depression Scale; MDI, Major Depression Inventory; GDS, Yesavage Geriatric Depression Scale; CSDD, Cornell Scale for Depression in Dementia; ATYP, the 8-item addendum of the Structured Interview Guide for the Hamilton Depression Rating Scale–Seasonal Affective Disorders Version Self-Rating Format*.

### Effect of Melatonin on Depressive Symptoms

**Figure 2:**
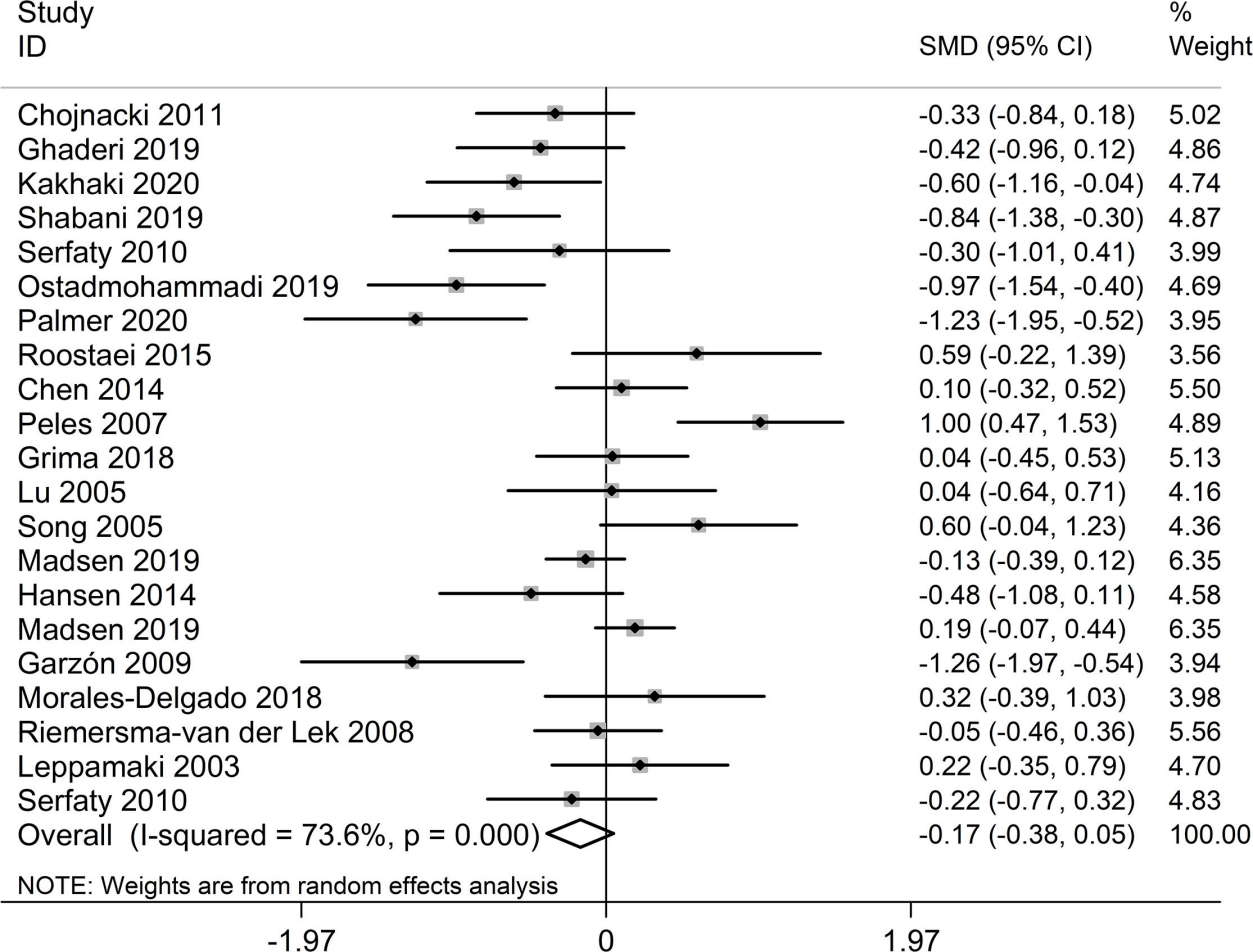
Meta-analysis on therapeutic effect of melatonin depression measured by eight scales. SMD, standardized mean difference; CI, confidence interval.

As shown in Figure 2 and Table 2, no significant results were observed in the overall meta-analysis of the effect of melatonin on depression treatment (SMD = −0.17, 95% CI = [−0.38, 0.05]). Significant heterogeneity was identified across the included studies (*I*^2^ = 73.6%, *p* <0.001). Furthermore, subgroup analyses on melatonin dosages and treatment durations were conducted, in which melatonin was shown to be significantly effective at the dosages of 10 mg/day (SMD = −0.70, 95% CI = [−0.98, −0.43]) and 20 mg/day (SMD = −1.23, 95% CI = [−1.95, −0.52]) (Supplementary Figure S1 and Table 2). However, no significant results were observed at other dosages, including 2, 2.5, 3, 5, 6, and 25 mg/day. As depicted in Supplementary Figure S2 and Table 2, a significance decrease in depression scores was found after 12 weeks of melatonin treatment (SMD = −0.41, 95% CI = [−0.69, −0.13]). Meanwhile, no positive results were observed after 1.5, 2, 3, 4, 5, 6, 8, and 48 weeks treatment.

**Table 2:** Subgroup analyses on the effect of melatonin on depressive symptoms.

**Subgroup**		**SMD (95% CI)**	***P***
Dosages	2 mg/day	0.12 (−0.25, 0.49)	0.536
	2.5 mg/day	−0.05 (−0.46, 0.36)	0.803
	3 mg/day	0.26 (−0.03, 0.55)	0.084
	5 mg/day	−0.05 (−0.97, 0.87)	0.909
	6 mg/day	−0.33 (−0.68, 0.02)	0.062
	10 mg/day	−0.70 (−0.98, −0.43)	<0.001
	20 mg/day	−1.23 (−1.95, −0.52)	0.001
	25 mg/day	0.03 (−0.29, 0.34)	0.896
Treatment durations	1.5 weeks	−1.23 (−1.95, −0.52)	0.001
	2 weeks	0.10 (−0.40, 0.61)	0.683
	3 weeks	0.22 (−0.35, 0.79)	0.449
	4 weeks	−0.08 (−0.44, 0.29)	0.680
	5 weeks	−0.30 (−1.01, 0.41)	0.404
	6 weeks	0.46 (−0.57, 1.49)	0.382
	8 weeks	−0.30 (−1.23, 0.64)	0.533
	12 weeks	−0.41 (−0.69, −0.13)	0.004
	48 weeks	0.59 (−0.22, 1.39)	0.153
Depression scales	BDI	−0.52 (−0.73, −0.31)	<0.001
	HADS-D	−0.01 (−0.22, 0.19)	0.900
	CES-D	0.53 (−0.35, 1.42)	0.235
	MDI	−0.09 (−0.74, 0.56)	0.777
	GDS	−0.47 (−2.01, 1.07)	0.551
	CSDD	−0.05 (−0.46, 0.36)	0.803
	ATYP	0.22 (−0.35, 0.79)	0.449
	HDRS-21	−0.22 (−0.77, 0.32)	0.424

*SMD, standardized mean difference; CI, confidence interval; BDI, Beck Depression Inventory; HADS-D, the Hospital Anxiety and Depression Scale; CES-D, Center for Epidemiological Studies Depression Scale; MDI, Major Depression Inventory; GDS, Yesavage Geriatric Depression Scale; CSDD, Cornell Scale for Depression in Dementia; ATYP, the 8-item addendum of the Structured Interview Guide for the Hamilton Depression Rating Scale–Seasonal Affective Disorders Version Self-Rating Format; HDRS-21, 21-item Hamilton Depression Rating Scale*.

### Effect of Melatonin on Depressive Symptoms as Measured by BDI

Eight studies reported depression severity measured using the BDI. As displayed in Supplementary Figure S3A and Table 2, a significant difference in BDI scores was identified between melatonin and placebo groups (SMD = −0.52, 95% CI = [−0.73, −0.31]) without significant bias induced by heterogeneity (*I*^2^ = 49.0%, *p* = 0.056). Subgroup analysis revealed that the SMD of BDI scores was −0.56 (95% CI = [−0.80, −0.32]) after 12 weeks of melatonin treatment compared with placebo (Supplementary Figure S3B and Table 2). Collectively, the results of three studies in which participants were treated with 10 mg of melatonin per day for 12 weeks showed that melatonin significantly alleviated depressive symptoms (SMD = −0.63, 95% CI = [−0.90, −0.35]) (Supplementary Figure S3C and Table 2). The above data indicate that the most effective strategy was to treat melatonin for 12 weeks with a daily dose of 10 mg.

### Effect of Melatonin on Depressive Symptoms as Measured by HADS-D

Four original studies used the HADS-D to evaluate depressive symptoms. As illustrated in Supplementary Figure S4 and Table 2, no significant decrease in HADS-D scores was observed between the melatonin and placebo groups (SMD = −0.01, 95% CI = [−0.22, 0.19]). Similar results were found in subgroup analysis using the treatment regimen of 3 mg/day melatonin (SMD = 0.33, 95% CI = [−0.13, 0.80]) (Supplementary Figure S5). No significant heterogeneity was observed across the above studies.

### Effect of Melatonin on Depressive Symptoms as Measured by CES-D

We synthesized two studies that measured depressive symptoms with the CES-D. As shown in Supplementary Figure S6 and Table 2, the pooled result reflected no significant effect of melatonin on CES-D scores (SMD = 0.53, 95% CI = [−0.35, 1.42]) with significant heterogeneity (*I*^2^ = 85.1%, *p* = 0.010).

### Effect of Melatonin on Depressive Symptoms as Measured by MDI

Two original studies used the MDI to evaluate depression severity. As listed in Supplementary Figure S7 and Table 2, no significant difference was observed between the melatonin and placebo groups (SMD = −0.09, 95% CI = [−0.74, 0.56]) with significant heterogeneity (*I*^2^ = 76.0%, *p* = 0.041).

### Effect of Melatonin on Depressive Symptoms as Measured by GDS

We included two studies that reported depression severity with the GDS. As Supplementary Figure S8 and Table 2 indicate, the pooled result demonstrated no significant effect of melatonin on GDS scores (SMD = −0.47, 95% CI = [−2.01, 1.07]) with significant heterogeneity (*I*^2^ = 89.3%, *p* = 0.002).

### Effect of Melatonin on Depressive Symptoms as Measured by HDRS-21, CSDD, or ATYP

Regarding other scales, only one study reported depressive symptoms with the HDRS-21, CSDD, or ATYP. As such, no meta-analysis was conducted.

### Evaluation of Risk of Bias According to the Cochrane Risk of Bias Tool

**Figure 3:**
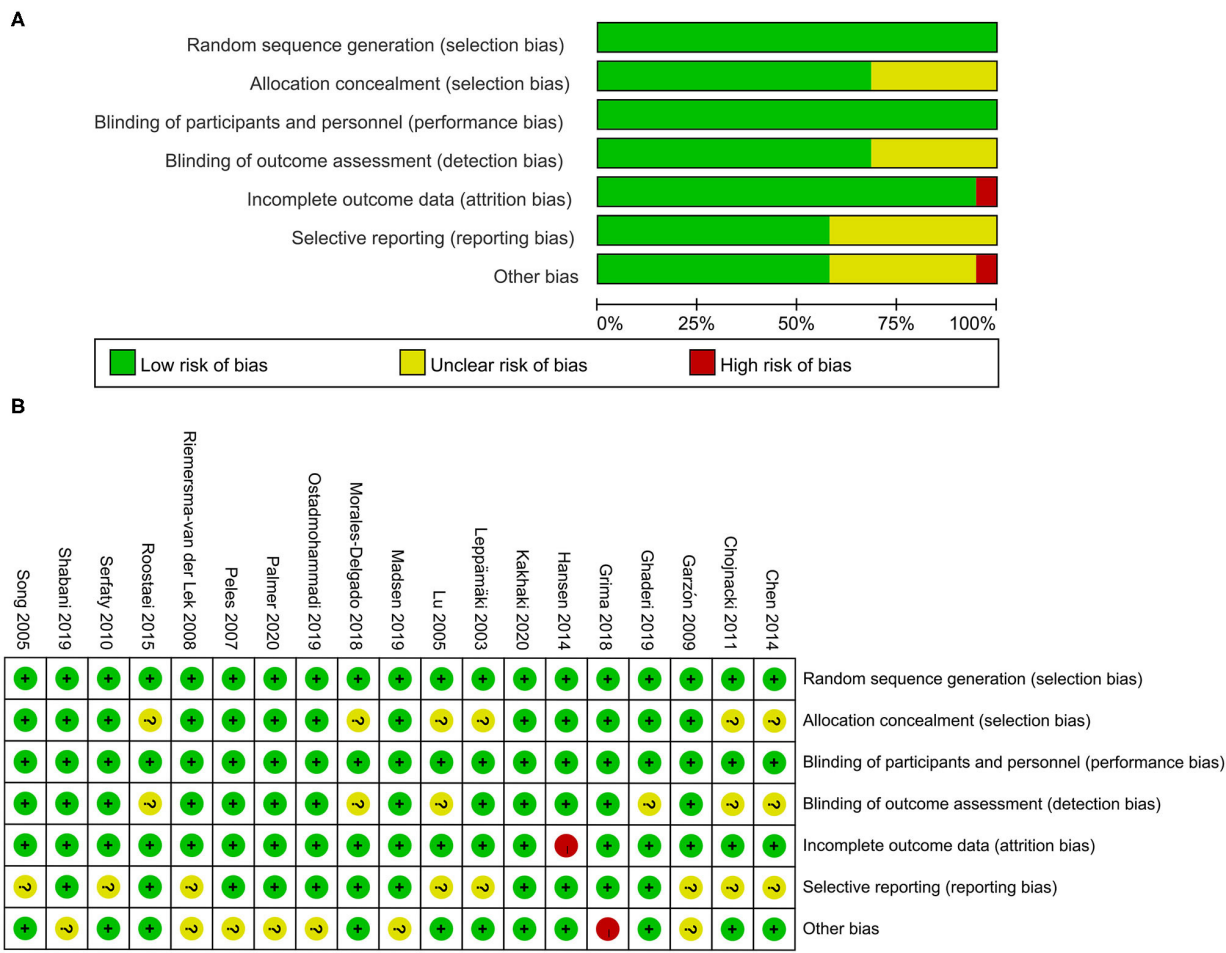
**(A,B)** Risk of bias graph and summary. Green means low risk of bias; yellow means unclear risk of bias; red means high risk of bias.

The risk of bias among included studies is presented in Figure 3. All studies had a low risk of bias on random sequence generation (i.e., selection bias) and blinding of participants and personnel (i.e., performance bias). The random assignment method and generation of random sequences were clarified. The application of blinding was described as well. Thirteen studies involved a low risk of bias on allocation concealment (i.e., selection bias); six other studies had an unclear risk. As for detection bias induced by a lack of blinding in outcome assessment, 13 studies had a low risk of bias while six had an unclear risk. Only one study had a high risk of attrition bias that might be caused by the uneven frequencies of participants lost to follow-up between the intervention and control groups. Eleven studies had a low bias on selective reporting (i.e., reporting bias) because these studies were carried out in accordance with the registered protocol; the other eight studies did not indicate the clinical trial registry in detail.

### Publication Bias and Sensitivity Analysis

**Figure 4:**
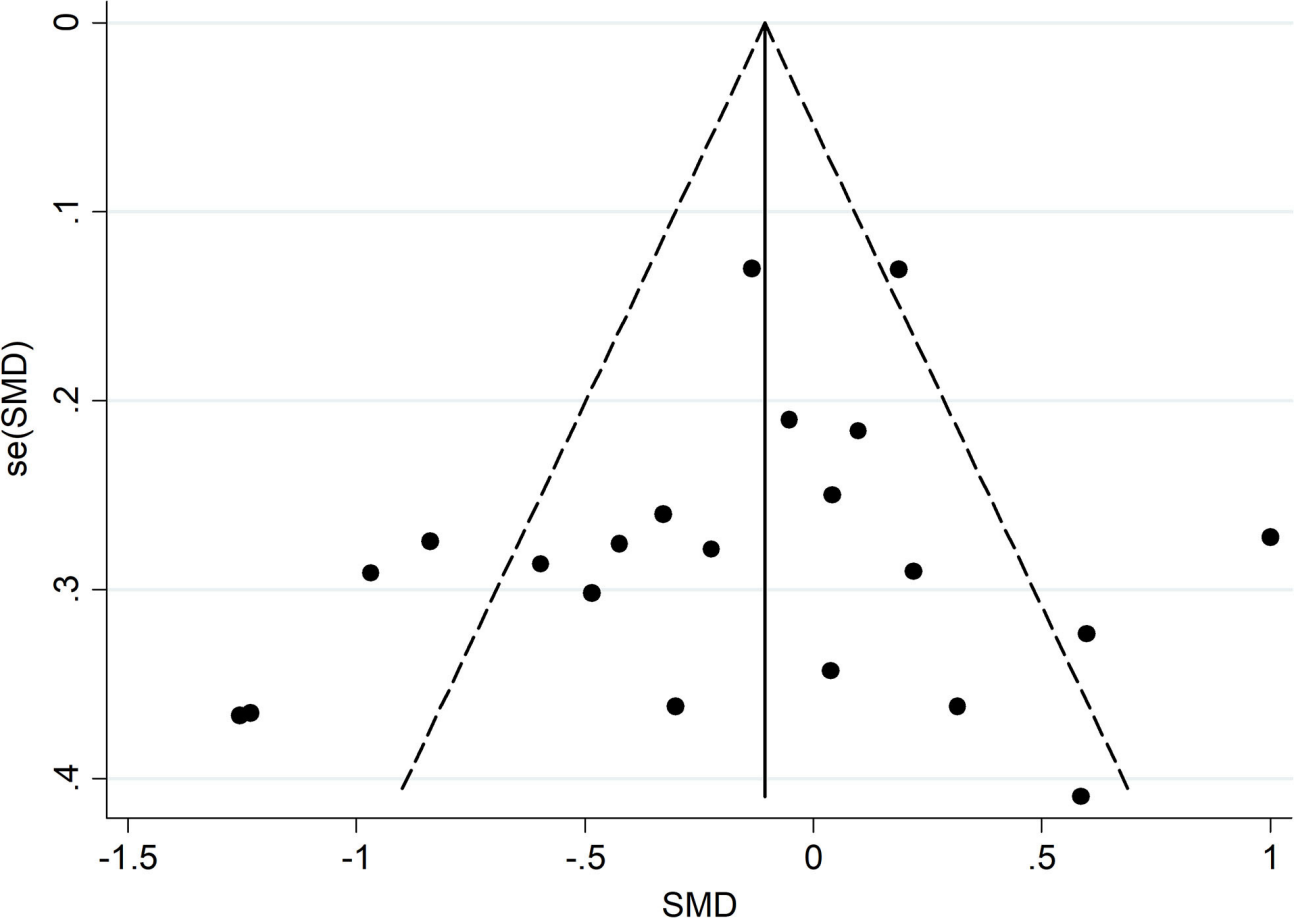
Funnel plot analysis. SMD, standardized mean difference; SE, standard error.

A funnel plot and Egger's test were used to confirm whether the meta-analysis results were influenced by publication bias. As presented in Figure 4, Supplementary Figures S9, S10, no significant bias emerged in meta-analysis of the combination of eight scales ($P_{Egger^{\prime }s}$ = 0.278), the BDI ($P_{Egger^{\prime }s}$ = 0.459), and the HADS-D ($P_{Egger^{\prime }s}$ = 0.219). Sensitivity analyses showed no substantial changes in the combined results when each study was removed in turn, highlighting the stability of our meta-analysis (Supplementary Figures S11–S13). Tests for the CES-D, MDI, and GDS were not carried out due to the small number of included studies.

### Adverse Effects of Melatonin

Eight of 19 studies (15, 16, 19, 21, 24, 28, 30, 34) discussed adverse events associated with melatonin administration (e.g., headache, daytime sleepiness, dizziness, poor sleep, insomnia, a fuzzy feeling, altered bowel habits, and tachycardia), among which six reported data on adverse events (15, 16, 19, 21, 28, 30). The average adverse event rate was 16.41% in the melatonin group and 14.73% in the placebo group. Meta-analysis showed no significant difference in adverse events between the melatonin and placebo groups (odds ratio = 1.52, 95% CI = [0.80, 2.91], *p* = 0.135) (Supplementary Figure S14).

## Discussion

Our systematic review and meta-analysis demonstrated that melatonin was not significantly effective for alleviating depressive symptoms. Although statistically positive results were observed at the dosages of 10 and 20 mg/day, there was very limited evidence for effects of melatonin on depression. Eight scales were involved in the measurement of depressive symptoms, among which the effect of melatonin was significant when the BDI was used. However, no significant effects were identified when depressive symptoms were measured with the HADS-D, CES-D, MDI, or GDS.

Depressive disorders are characterized by psychological, behavioral, and physiological changes, affecting 17–20% of the population globally (36). Circadian rhythm disturbance is a major feature of depressive symptoms: 70% of patients with severe depression suffer from sleep disturbances (37, 38). The association between depression and desynchronized circadian rhythms suggests that agents improving circadian rhythms may contribute to relieving depression. Melatonin, an endogenous hormone mainly synthesized in the pineal gland, plays a key role in synchronizing circadian rhythms. By binding to two receptors (MT_1_ and MT_2_) in the suprachiasmatic nucleus, melatonin regulates the expression of clock genes, resulting in a chronobiotic effect (26, 37, 39). Patients with depression have shown reduced peak values of plasma melatonin at night with a disordered rhythm phase (40). Exogenous melatonin also has an effect on synchronizing rhythms; it is thus thought to hold promise for treating depressive symptoms (38, 41–43). Furthermore, sleep disorders and biological rhythm abnormalities among individuals with depression are generally affected by sex. Women are twice as likely as men to suffer from depression due to the influence of gonadal hormones, which are involved in sleep regulation and human neurotransmitter regulation (44).

No biomarkers have yet been identified for the evaluation of depression; rather, some scales and questionnaires have been developed. The BDI is commonly used and consists of 21 items to assess cognitive, affective, behavioral, and somatic symptoms of depression (45, 46). And studies found that exogenous melatonin could lead to modulation in specific physiological functions like control of human mood, behavior, cognition, and sleep regulation by activating membrane MT_1_/MT_2_ melatonin receptors (47–49). In practice, the BDI is used not only to assess depression in psychiatric patients but also to screen for depressive symptoms in various patient groups (50, 51), and most participants in this meta-analysis suffered secondary depression from different diseases. Moreover, the BDI provides information on a relatively wide range of levels of depression, and is typically considered a standard tool for depression measurement with high accuracy and efficiency (45, 52). In terms of other scales, the HADS-D consists of seven questions and is often used with patients with moderate depression (53, 54). The HADS-D is designed to evaluate the anxiety and depressive symptoms of patients without physical symptoms (e.g., sleep disorders) (55). However, this scale provides less information than others (45). By contrast, the 10-item MDI is more accurate for assessing major (i.e., moderate to severe) depression (56). The CES-D, composed of 20 items, is also used in the general population and primary healthcare institutions (57). Yet, the CES-D is considered more appropriate for evaluating general distress than depression (58). The GDS is a self-rated scale used primarily to assess depression in older adults (35). In our sample, the effect of melatonin was found statistically positive when using the BDI, but not significant when using the other four depression scales (i.e., HADS-D, CES-D, MDI, and GDS).

As a medical sleep aid, a single dose of 1–10 mg of melatonin has is considered standard, but the optimal dose for depression is not yet known (59). Based on our findings, the dose of 10 mg/day might work more efficiently. However, no significant results were observed at other dosages, including 2, 2.5, 3, 5, 6, and 25 mg/day. In addition, studies have reported that 10 mg per day is a balanced option considering that melatonin is a safe, non-toxic drug with minor adverse events compared to placebo in long-term treatment (15, 16). With regard to treatment duration, no consistent program has been established for psychiatric use (60). Our subgroup analysis implied that 12-week treatment was more effective in alleviating depression, in line with the duration for melatonin receptor agonists agomelatine and ramelteon (61–63). However, no significant results were observed after 1.5, 2, 3, 4, 5, 6, 8, and 48 weeks treatment. This indicated that there is very limited evidence for effects of melatonin on depression.

No obvious side effects of melatonin were found in all original studies included in this systematic review. Some studies also showed that melatonin had no side effects in the short term and had mild side effects in the long term (59, 64).

### Limitations

Some limitations should be noted in our study. First, eight scales were used to measure depressive symptoms, which led to variation. Differences in these scales, as well as in participants' demographics and comorbidity, likely resulted in inconsistent outcomes between studies. Second, the number of studies included in our meta-analysis was small and might restrict the generalizability of our findings. Differences in melatonin dosage, treatment duration, and baseline treatment influenced the consolidation of our results. Finally, on the basis of studies included in meta-analysis, we cannot identify the specific type of depression on which melatonin may be effective than others.

## Conclusions

In conclusion, no sufficient results were obtained to evidence the effects of melatonin on depression. Melatonin might have no potential therapeutic effect on depression.

## Data Availability Statement

The original contributions presented in the study are included in the article/Supplementary Material, further inquiries can be directed to the corresponding author/s.

## Author Contributions

MN, YZ, and WZ supervised and designed this study. CL, ML, and DaM participated in the design and planning process. CL and DaM extracted and analyzed data and wrote the first draft of the manuscript. ML, TW, XZ, YH, and DaM checked the extracted and analyzed data. ML, EA, MN, YZ, and WZ revised the manuscript. All authors had access to all data in the study and are responsible for the integrity of the data and the accuracy of the analysis. All authors contributed to article and approved the final manuscript.

## Funding

This study was supported by the Natural Science Foundation of Shandong Province, China (ZR2017MH100), European Commission Horizon 2020 (PRODEMOS-779238), and National Key Research and Development Program in China (2017YFE0118800).

## Conflict of Interest

The authors declare that the research was conducted in the absence of any commercial or financial relationships that could be construed as a potential conflict of interest.

## Publisher's Note

All claims expressed in this article are solely those of the authors and do not necessarily represent those of their affiliated organizations, or those of the publisher, the editors and the reviewers. Any product that may be evaluated in this article, or claim that may be made by its manufacturer, is not guaranteed or endorsed by the publisher.

## Supplementary Material

Supplementary Figure S1 Subgroup analysis on dosage. SMD, standardized mean difference; CI, confidence interval.

Supplementary Figure S2 Subgroup analysis on treatment duration. SMD, standardized mean difference; CI, confidence interval.

Supplementary Figure S3 Meta-analysis of effect of melatonin on depression measured by BDI score. **(A)** Overall meta-analysis; **(B)** subgroup analysis of studies treated for 12 weeks by all dosages of melatonin; **(C)** subgroup analysis of studies treated for 12 weeks by 10 mg/day melatonin; BDI, Beck Depression Inventory; SMD, standardized mean difference; CI, confidence interval.

Supplementary Figure S4 Meta-analysis of effect of melatonin on depression measured by HADS-D score. HADS-D, the Hospital Anxiety and Depression Scale; SMD, standardized mean difference; CI, confidence interval.

Supplementary Figure S5 Subgroup meta-analysis on HADS-D score within patients treated by 3 mg/day melatonin. HADS-D, the Hospital Anxiety and Depression Scale; SMD, standardized mean difference; CI, confidence interval.

Supplementary Figure S6 Meta-analysis on CES-D score. CES-D, Center for Epidemiological Studies Depression Scale; SMD, standardized mean difference; CI, confidence interval.

Supplementary Figure S7 Meta-analysis on MDI score. MDI, Major Depression Inventory; SMD, standardized mean difference; CI, confidence interval.

Supplementary Figure S8 Meta-analysis on GDS score. GDS, Yesavage Geriatric Depression Scale; SMD, standardized mean difference; CI, confidence interval.

Supplementary Figure S9 Funnel plot analysis on BDI score. SMD, standardized mean difference; SE, standard error.

Supplementary Figure S10 Funnel plot analysis on HADS-D score. SMD, standardized mean difference; SE, standard error.

Supplementary Figure S11 Sensitivity analysis on all scales.

Supplementary Figure S12 Sensitivity analysis on BDI score.

Supplementary Figure S13 Sensitivity analysis on HADS-D score.

Supplementary Figure S14 Adverse event analysis. OR, Odds Ratio; CI, confidence interval.

Supplementary Table S1 Checklist of the Preferred Reporting Items for Systematic Reviews and Meta-Analyses (PRISMA). Y, the item was reported in article; N, the item was not reported.

Supplementary Box 1 Study protocol.
