# Supplementary material for: The Therapeutic Effect of Exogenous Melatonin on Depressive Symptoms: A Systematic Review and Meta-Analysis
Source: Front Psychiatry. 2022 Mar 17;13:737972. doi: 10.3389/fpsyt.2022.737972 (PMC8968118; doi:10.3389/fpsyt.2022.737972)
Supplement: Supplementary Figure S1 — Subgroup analysis on dosage. SMD, standardized mean difference; CI, confidence interval. [file Data_Sheet_1.docx]

**[Supplementary tables]**

**Table S1** Checklist of the Preferred Reporting Items for Systematic Reviews and Meta-Analyses (PRISMA)

| **Section/Topic** | **Checklist Item** | **Reported or not** |
| --- | --- | --- |
| TITLE |  |  |
| Title | Identify the report as a systematic review, meta-analysis, or both. | Y |
| ABSTRACT |  |  |
| Structured summary | Provide a structured summary including, as applicable: background; objectives; data sources; study eligibility criteria, participants, and interventions; study appraisal and synthesis methods; results; limitations; conclusions and implications of key findings; systematic review registration number. | Y |
| INTRODUCTION |  |  |
| Rationale | Describe the rationale for the review in the context of what is already known. | Y |
| Objectives | Provide an explicit statement of questions being addressed with reference to participants, interventions, comparisons, outcomes, and study design (PICOS). | Y |
| METHODS |  |  |
| Protocol and registration | Indicate if a review protocol exists, if and where it can be accessed (e.g., Web address), and, if available, provide registration information including registration number. | N |
| Eligibility criteria | Specify study characteristics (e.g., PICOS, length of follow-up) and report characteristics (e.g., years considered, language, publication status) used as criteria for eligibility, giving rationale. | Y |
| Information sources | Describe all information sources (e.g., databases with dates of coverage, contact with study authors to identify additional studies) in the search and date last searched. | Y |
| Search | Present full electronic search strategy for at least one database, including any limits used, such that it could be repeated. | Y |
| Study selection | State the process for selecting studies (i.e., screening, eligibility, included in systematic review, and, if applicable, included in the meta-analysis). | Y |
| Data collection process | Describe method of data extraction from reports (e.g., piloted forms, independently, in duplicate) and any processes for obtaining and confirming data from investigators. | Y |
| Data items | List and define all variables for which data were sought (e.g., PICOS, funding sources) and any assumptions and simplifications made. | Y |
| Risk of bias in individual studies | Describe methods used for assessing risk of bias of individual studies (including specification of whether this was done at the study or outcome level), and how this information is to be used in any data synthesis. | Y |
| Summary measures | State the principal summary measures (e.g., risk ratio, difference in means). | Y |
| Synthesis of results | Describe the methods of handling data and combining results of studies, if done, including measures of consistency (e.g., I2) for each meta-analysis. | Y |
| Risk of bias across studies | Specify any assessment of risk of bias that may affect the cumulative evidence (e.g., publication bias, selective reporting within studies). | Y |
| Additional analyses | Describe methods of additional analyses (e.g., sensitivity or subgroup analyses, meta-regression), if done, indicating which were pre-specified. | Y |
| RESULTS |  |  |
| Study selection | Give numbers of studies screened, assessed for eligibility, and included in the review, with reasons for exclusions at each stage, ideally with a flow diagram. | Y |
| Study characteristics | For each study, present characteristics for which data were extracted (e.g., study size, PICOS, follow-up period) and provide the citations. | Y |
| Risk of bias within studies | Present data on risk of bias of each study and, if available, any outcome-level assessment (see Item 12). | Y |
| Results of individual studies | For all outcomes considered (benefits or harms), present, for each study: (a) simple summary data for each intervention group and (b) effect estimates and confidence intervals, ideally with a forest plot. | Y |
| Synthesis of results | Present results of each meta-analysis done, including confidence intervals and measures of consistency. | Y |
| Risk of bias across studies | Present results of any assessment of risk of bias across studies (see Item 15). | Y |
| Additional analysis | Give results of additional analyses, if done (e.g., sensitivity or subgroup analyses, meta-regression [see Item 16]). | Y |
| DISCUSSION |  | Y |
| Summary of evidence | Summarize the main findings including the strength of evidence for each main outcome; consider their relevance to key groups (e.g., health care providers, users, and policy makers). | Y |
| Limitations | Discuss limitations at study and outcome level (e.g., risk of bias), and at review level (e.g., incomplete retrieval of identified research, reporting bias). | Y |
| Conclusions | Provide a general interpretation of the results in the context of other evidence, and implications for future research. | Y |
| FUNDING |  |  |
| Funding | Describe sources of funding for the systematic review and other support (e.g., supply of data) | Y |

Y: the item was reported in article, N: the item was not reported.

**[Supplementary box]**

**Box 1** Study protocol

| 1. Review title.   Give the title of the review in English: The therapeutic effect of exogenous melatonin on depressive symptoms: A systematic review and meta-analysis.   1. Anticipated or actual start date.   Give the date the systematic review started or is expected to start: 10/02/2021   1. Review question.   We used restriction options in accordance with the Population, Intervention, Comparison, and Outcomes principle: study participants were selected from an adult population; the intervention agent was melatonin; the comparison agent was placebo; and the outcome was the relevant score for depressive symptoms as measured by investigators.   1. Searches.   We searched PubMed, EMBASE, and the Cochrane Library for trials published up to May 15, 2021. The terms “melatonin, depression, depressive disorders, mood disorders, depressive symptoms, treat, effect, and therapeutics” were used for the literature search. The search strategies are as follows:  (1) PubMed: (((("Depression"[MeSH Major Topic]) OR (Depressions) OR (Depressive Symptoms) OR (Depressive Symptom) OR (Symptom, Depressive) OR (Symptoms, Depressive) OR (Emotional Depression) OR (Depression, Emotional) OR (Depressions, Emotional) OR (Emotional Depressions)) OR (("Depressive Disorder"[MeSH Major Topic]) OR (Depressive Disorders) OR (Disorder, Depressive) OR (Disorders, Depressive) OR (Neurosis, Depressive) OR (Depressive Neuroses) OR (Depressive Neurosis) OR (Neuroses, Depressive) OR (Depression, Endogenous) OR (Depressions, Endogenous) OR (Endogenous Depression) OR (Endogenous Depressions) OR (Depressive Syndrome) OR (Depressive Syndromes) OR (Syndrome, Depressive) OR (Syndromes, Depressive) OR (Depression, Neurotic) OR (Depressions, Neurotic) OR (Neurotic Depression) OR (Neurotic Depressions) OR (Melancholia) OR (Melancholias) OR (Unipolar Depression) OR (Depression, Unipolar) OR (Depressions, Unipolar) OR (Unipolar Depressions)) OR (("mood Disorders"[MeSH Major Topic]) OR (Disorder, Mood) OR (Disorders, Mood) OR (Mood Disorder) OR (Affective Disorders) OR (Affective Disorder) OR (Disorder, Affective) OR (Disorders, Affective))) AND (("melatonin"[MeSH Major Topic]) OR (melatonin)) AND (effect* OR treat* OR therapeutics));  (2) EMBASE: (melatonin:ab,ti AND (depression:ab,ti OR 'emotional depressions':ab,ti OR 'depressions, emotional':ab,ti OR 'depression, emotional':ab,ti OR 'emotional depression':ab,ti OR 'symptoms, depressive':ab,ti OR 'symptom, depressive':ab,ti OR 'depressive symptom':ab,ti OR 'disorders, affective':ab,ti OR 'disorder, affective':ab,ti OR 'affective disorder':ab,ti OR 'affective disorders':ab,ti OR 'mood disorder':ab,ti OR 'disorders, mood':ab,ti OR 'disorder, mood':ab,ti OR 'unipolar depressions':ab,ti OR 'depressions, unipolar':ab,ti OR 'depression, unipolar':ab,ti OR 'unipolar depression':ab,ti OR 'melancholias':ab,ti OR 'melancholia':ab,ti OR 'neurotic depressions':ab,ti OR 'neurotic depression':ab,ti OR 'depressions, neurotic':ab,ti OR 'depression, neurotic':ab,ti OR 'syndromes, depressive':ab,ti OR 'syndrome, depressive':ab,ti OR 'depressive syndromes':ab,ti OR 'depressive syndrome':ab,ti OR 'endogenous depressions':ab,ti OR 'endogenous depression':ab,ti OR 'depressions, endogenous':ab,ti OR 'depression, endogenous':ab,ti OR 'neuroses, depressive':ab,ti OR 'depressive neurosis':ab,ti OR 'depressive neuroses':ab,ti OR 'neurosis, depressive':ab,ti OR 'disorder, depressive':ab,ti OR 'disorders, depressive':ab,ti OR 'depressive disorders':ab,ti OR 'mood disorders':ab,ti OR 'depressive symptoms':ab,ti) AND (effect*:ab,ti OR therapeutics:ab,ti OR treat*:ab,ti) AND ([controlled clinical trial]/lim OR [randomized controlled trial]/lim) AND ([adult]/lim OR [aged]/lim) AND [humans]/lim AND [embase]/lim);  (3) the Cochrane Library: (((depression):ti,ab,kw OR (depressive disorders):ti,ab,kw OR (depressive symptoms):ti,ab,kw OR (mood disorders):ti,ab,kw OR (MeSH descriptor: [Depression] this term only and with qualifier(s): [drug therapy - DT]) OR (MeSH descriptor: [Depression] this term only and with qualifier(s): [prevention & control - PC]) OR (MeSH descriptor: [Depressive disorder] this term only) OR (MeSH descriptor: [Mood disorders] this term only)) AND ((MeSH descriptor: [Melatonin] explode all trees and with qualifier(s): [adverse effects - AE]) OR (MeSH descriptor: [Melatonin] explode all trees and with qualifier(s): [therapeutic use - TU]) OR (melatonin):ti,ab,kw) AND ((effect*):ti,ab,kw OR (therapeutics):ti,ab,kw OR (treat*):ti,ab,kw))   1. Condition or domain being studied.   Give a short description of the disease, condition or healthcare domain being studied in your systematic review. Depression, one of the most common psychiatric disorders, contributes to morbidity and mortality in adults and adolescents worldwide.   1. Participants/population.   Inclusion criteria: study participants were adults (The age requirement is 18 years or older).   1. Intervention(s), exposure(s).   Inclusion criteria: the effect of melatonin on remission of depression was investigated among participants with depressive symptoms; the study involved the diagnosis and/or measurement of depression; the severity of depression was rated by a self- or clinician-administered questionnaire; the mean severity of depressive  symptoms was compared between melatonin and placebo groups.  Exclusion criteria: melatonin was not given as an intervention; no mean or median score was reported on the depression measurement in the melatonin or placebo groups; the effect of melatonin or placebo was not isolated from other interventions.  8. Comparator(s)/control.  Inclusion criteria: the mean severity of depressive symptoms was compared between melatonin and placebo groups.  Exclusion criteria: no mean or median score was reported on the depression measurement in the melatonin or placebo groups; the effect of melatonin or placebo was not isolated from other interventions.  9. Types of study to be included.  Inclusion criteria: RCTs or randomized crossover trials were performed.  10. Main outcome.  To clarify the effectiveness of melatonin for depression  11. Data extraction.  The authors plan to extract data from the included studies, such as the study characteristics, primary outcomes, measurement instruments, and adverse events. For studies that provide the mean and standard deviation (SD) of depression scores, the authors will record summary data directly, otherwise we calculated summary data using statistical approaches.  12. Risk of bias (quality) assessment.  Evaluation of risk of bias according to the Cochrane risk of bias tool.  13. Strategy for data synthesis.  The meta-analysis will conduct in STATA 14.0 software (Stata Corp, College Station, TX, USA). Meta-analysis was performed on each scale separately. The standardized mean difference (SMD) and 95% CI were synthesized to evaluate the difference in depression scores between the melatonin and placebo groups. Statistical heterogeneity was examined on the basis of Cochran’ s Q test and the I² statistic. Sensitivity analysis was conducted by removing each original study one at a time. Publication bias was identified through funnel plot analysis along with Egger’s regression asymmetry test.  14. Analysis of subgroups or subsets.  Subgroup analyses were performed on the basis of melatonin dosage, treatment duration and/or different scales.  15. Keywords.  Key words: Melatonin; Depression; Depressive symptoms; Therapeutic effect; Systematic review |
| --- |

**[Supplementary figures]**


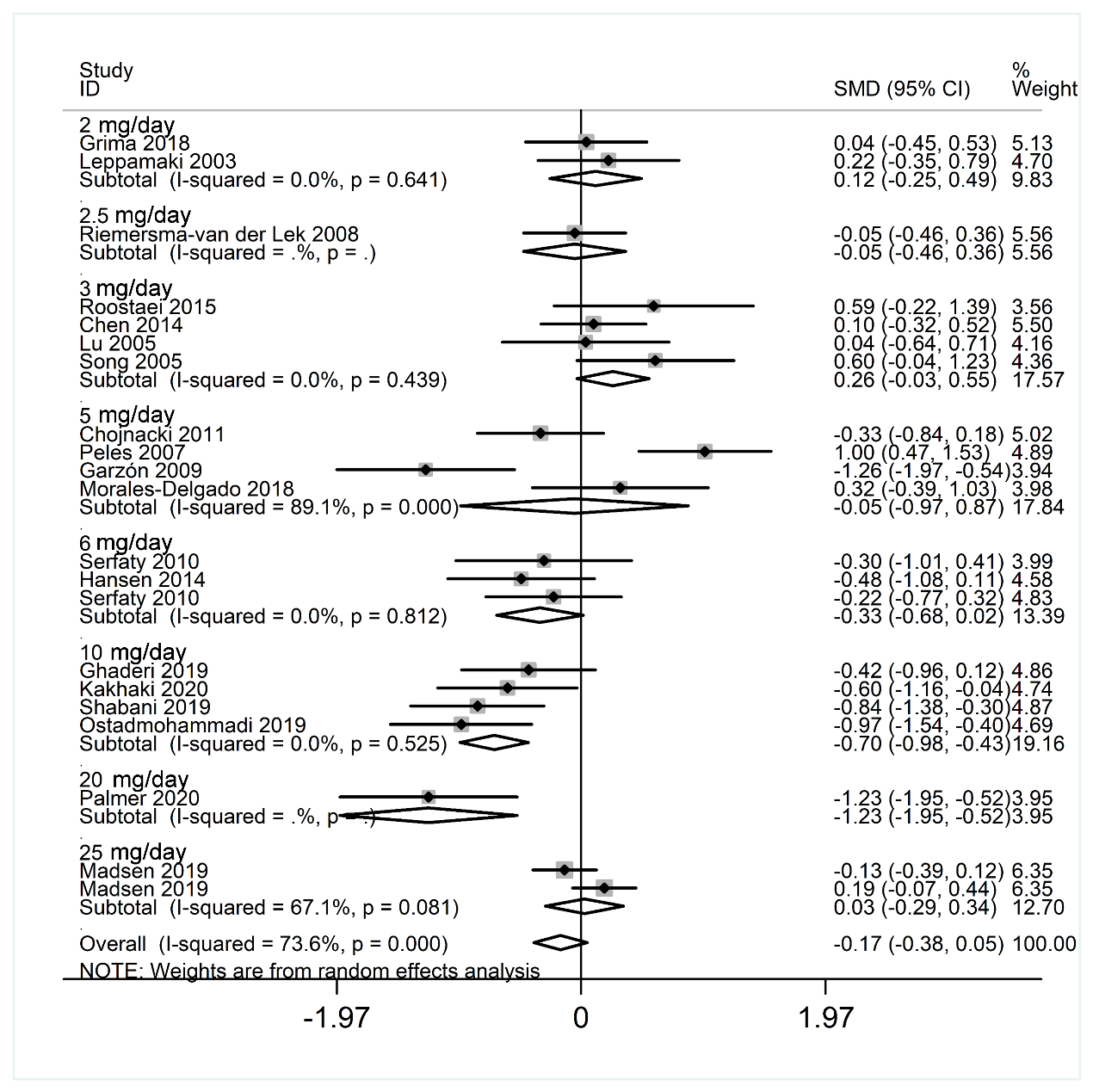


**Figure S1** Subgroup analysis on dosage. SMD, standardized mean difference; CI, confidence interval


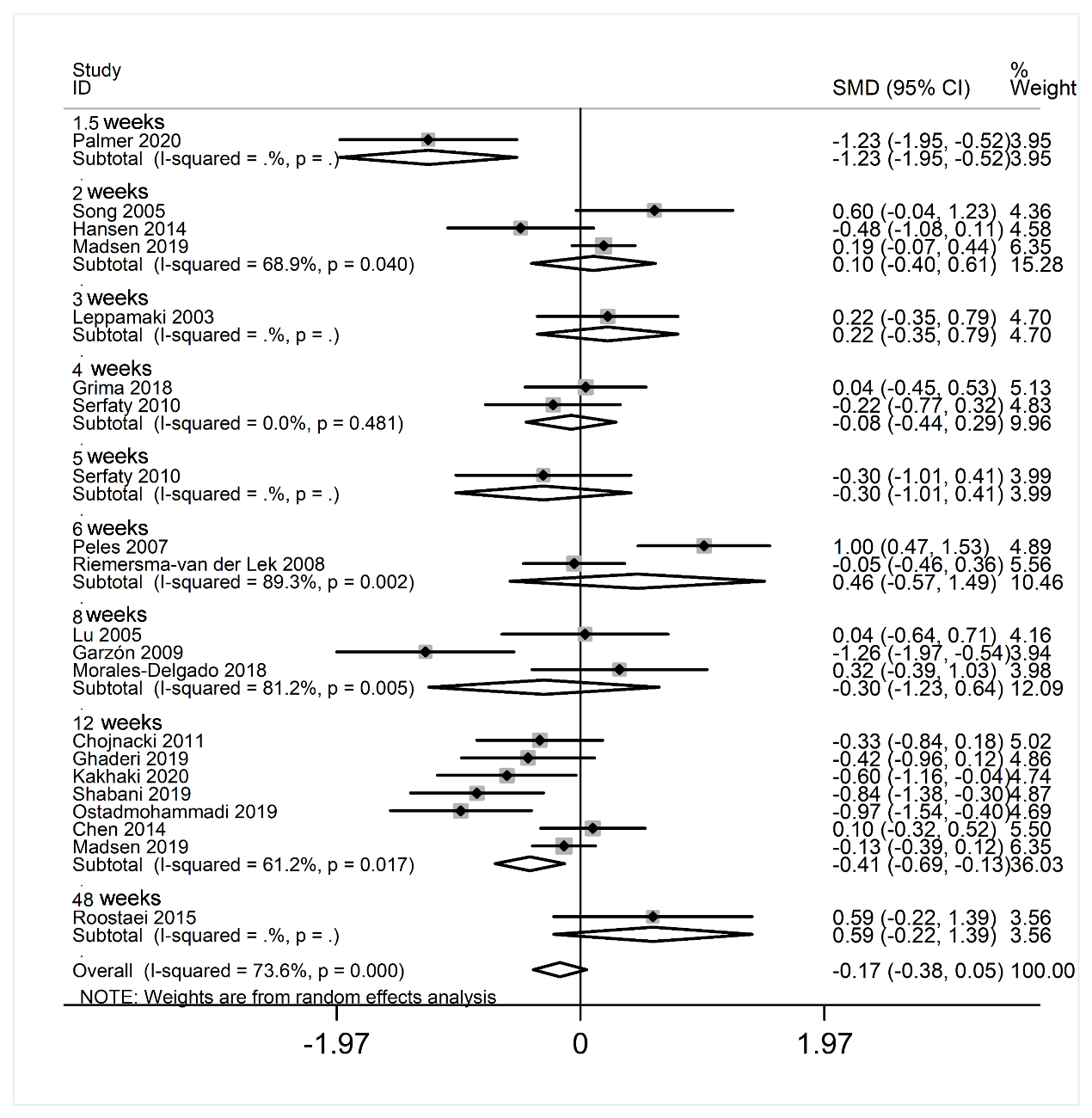


**Figure S2** Subgroup analysis on treatment duration. SMD, standardized mean difference; CI, confidence interval


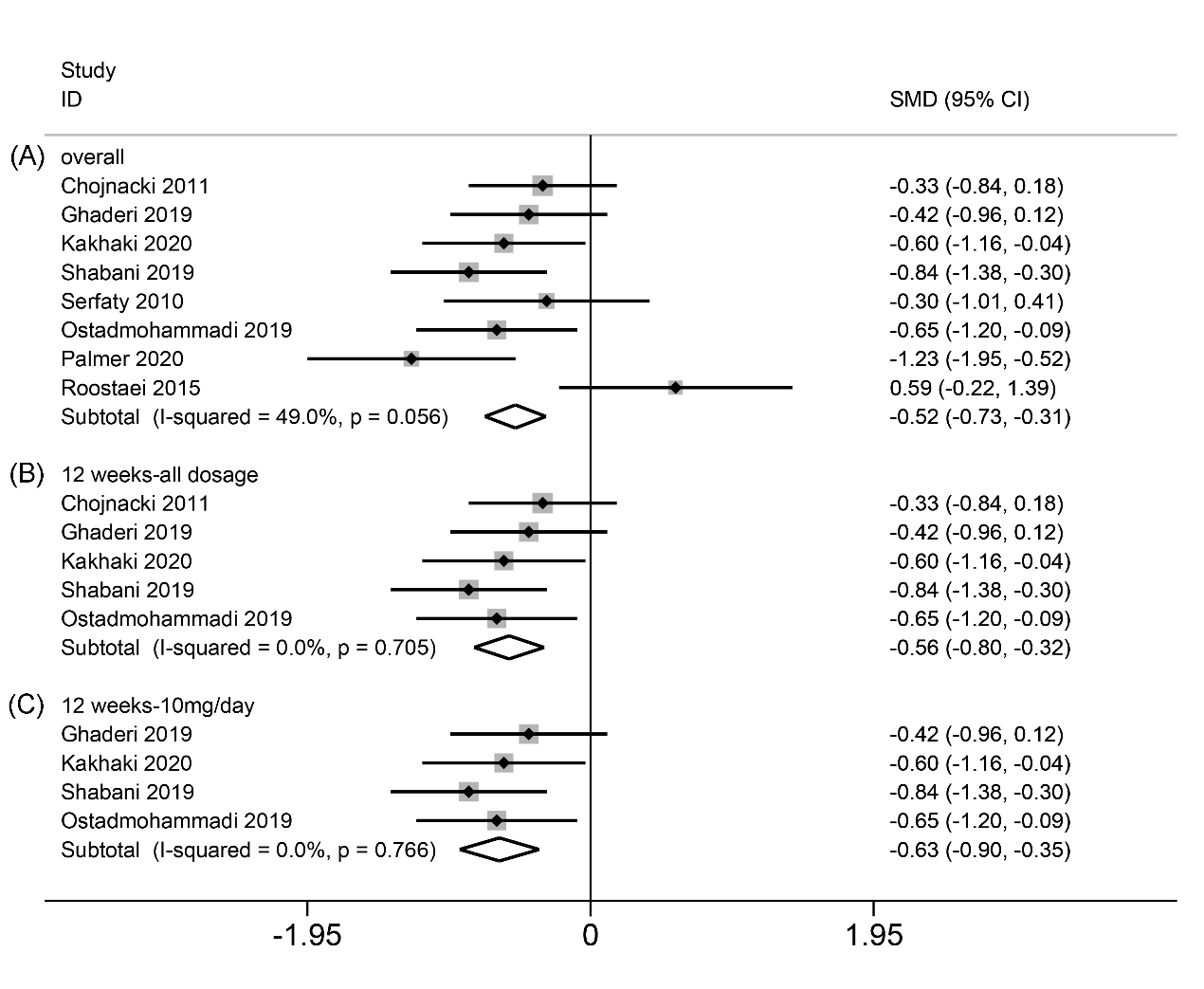


**Figure S3** Meta-analysis of effect of melatonin on depression measured by BDI score. (A) Overall meta-analysis; (B) Subgroup analysis of studies treated for 12 weeks by all dosages of melatonin; (C) Subgroup analysis of studies treated for 12 weeks by 10 mg/day melatonin; BDI, Beck Depression Inventory; SMD, standardized mean difference; CI, confidence interval


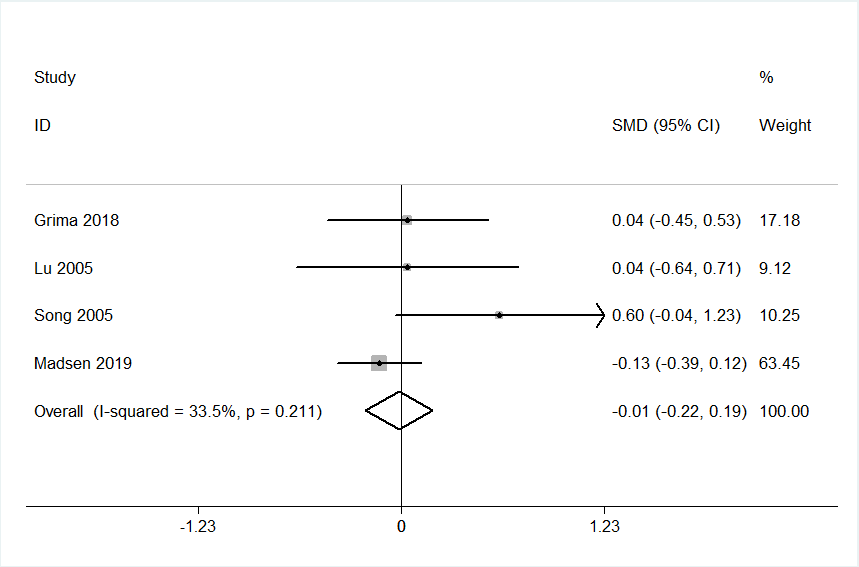


**Figure S4** Meta-analysis of effect of melatonin on depression measured by HADS-D score. HADS-D, the Hospital Anxiety and Depression Scale; SMD, standardized mean difference; CI, confidence interval


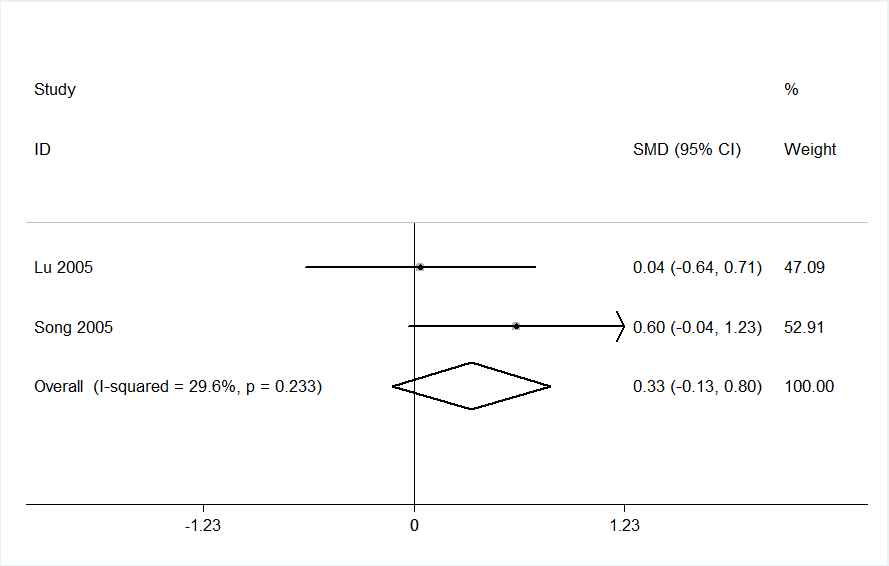


**Figure S5** Subgroup meta-analysis on HADS-D score within patients treated by 3 mg/day melatonin. HADS-D, the Hospital Anxiety and Depression Scale; SMD, standardized mean difference; CI, confidence interval


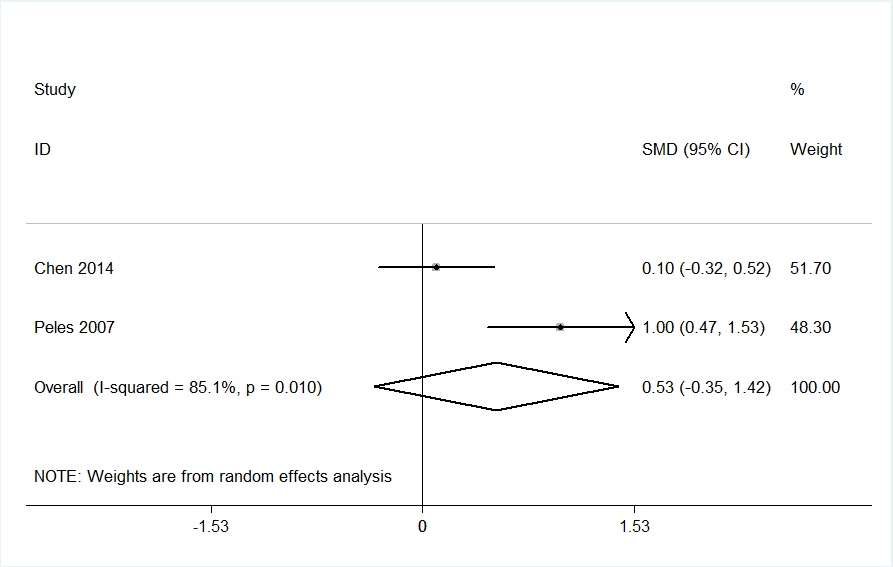


**Figure S6** Meta-analysis on CES-D score. CES-D: Center for Epidemiological Studies Depression Scale; SMD, standardized mean difference; CI, confidence interval


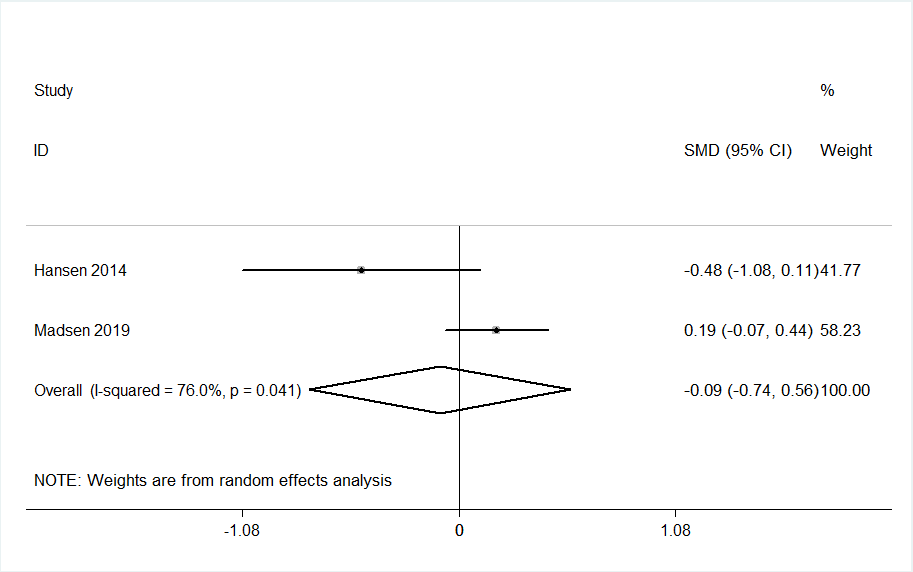


**Figure S7** Meta-analysis on MDI score. MDI: Major Depression Inventory; SMD, standardized mean difference; CI, confidence interval


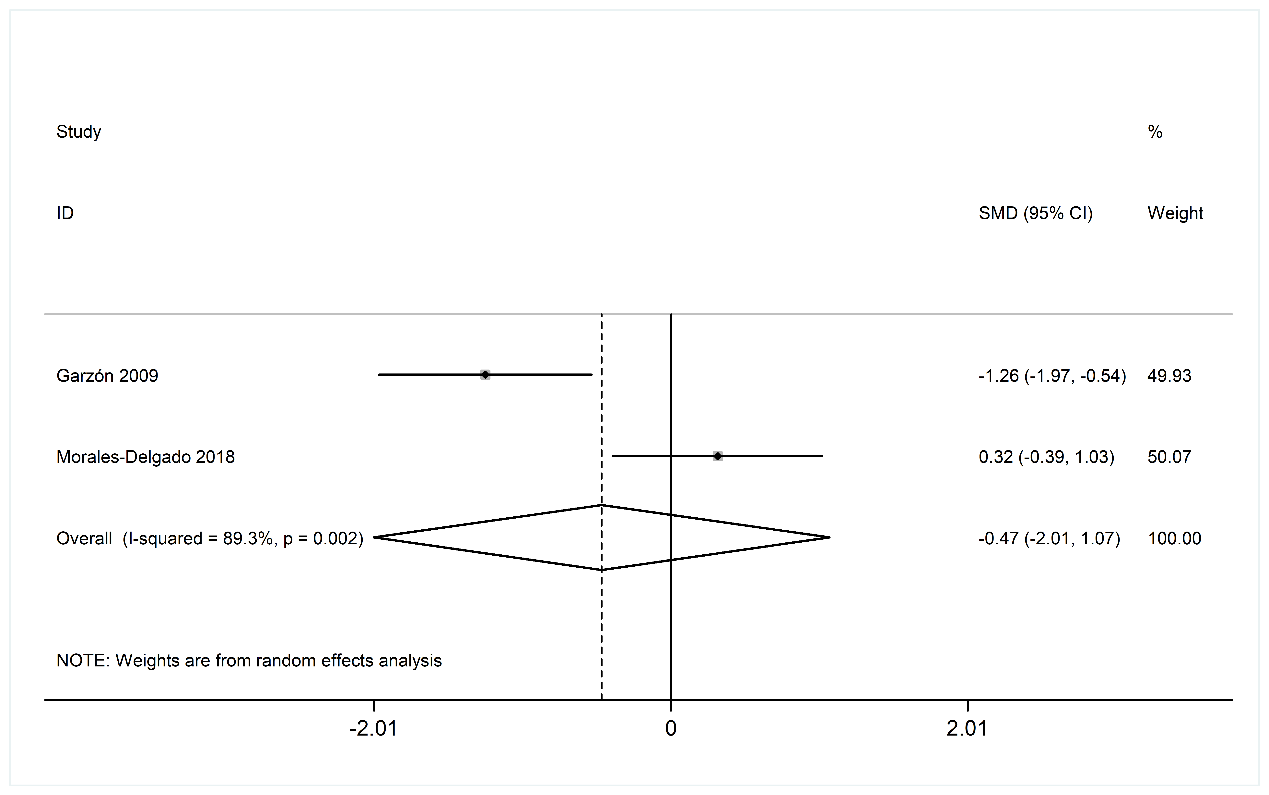


**Figure S8** Meta-analysis on GDS score. GDS: Yesavage Geriatric Depression Scale; SMD, standardized mean difference; CI, confidence interval


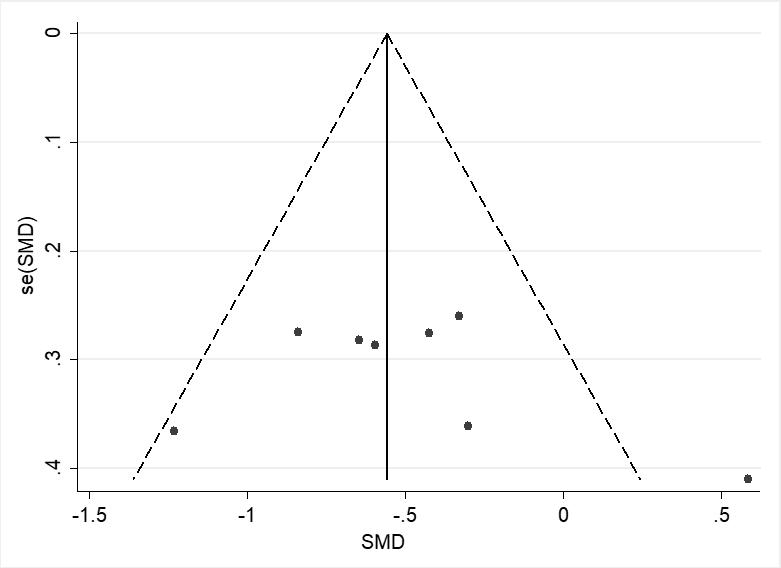


**Figure S9** Funnel plot analysis on BDI score. SMD, standardized mean difference; SE, standard error

**Figure S10** Funnel plot analysis on HADS-D score. SMD, standardized mean difference; SE, standard error


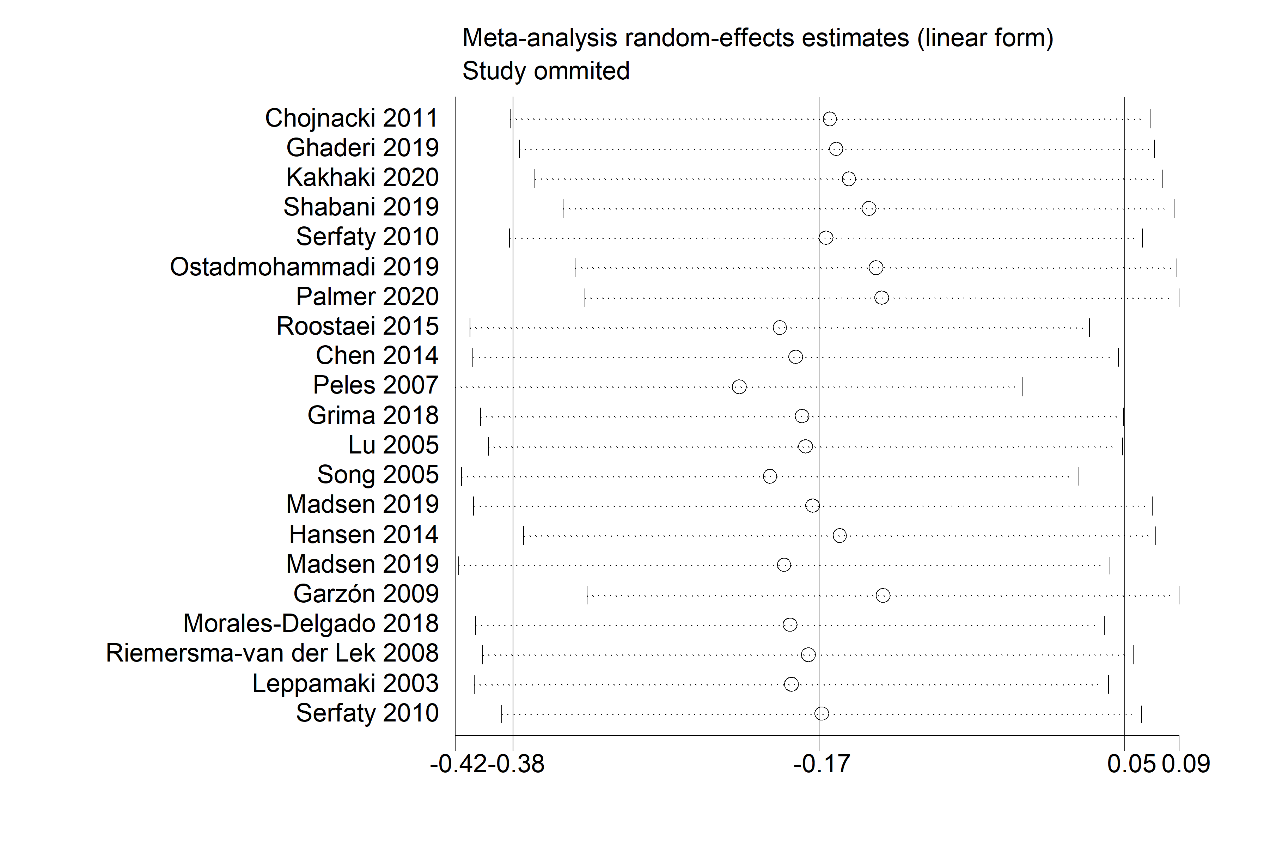


**Figure S11** Sensitivity analysis on all scales.


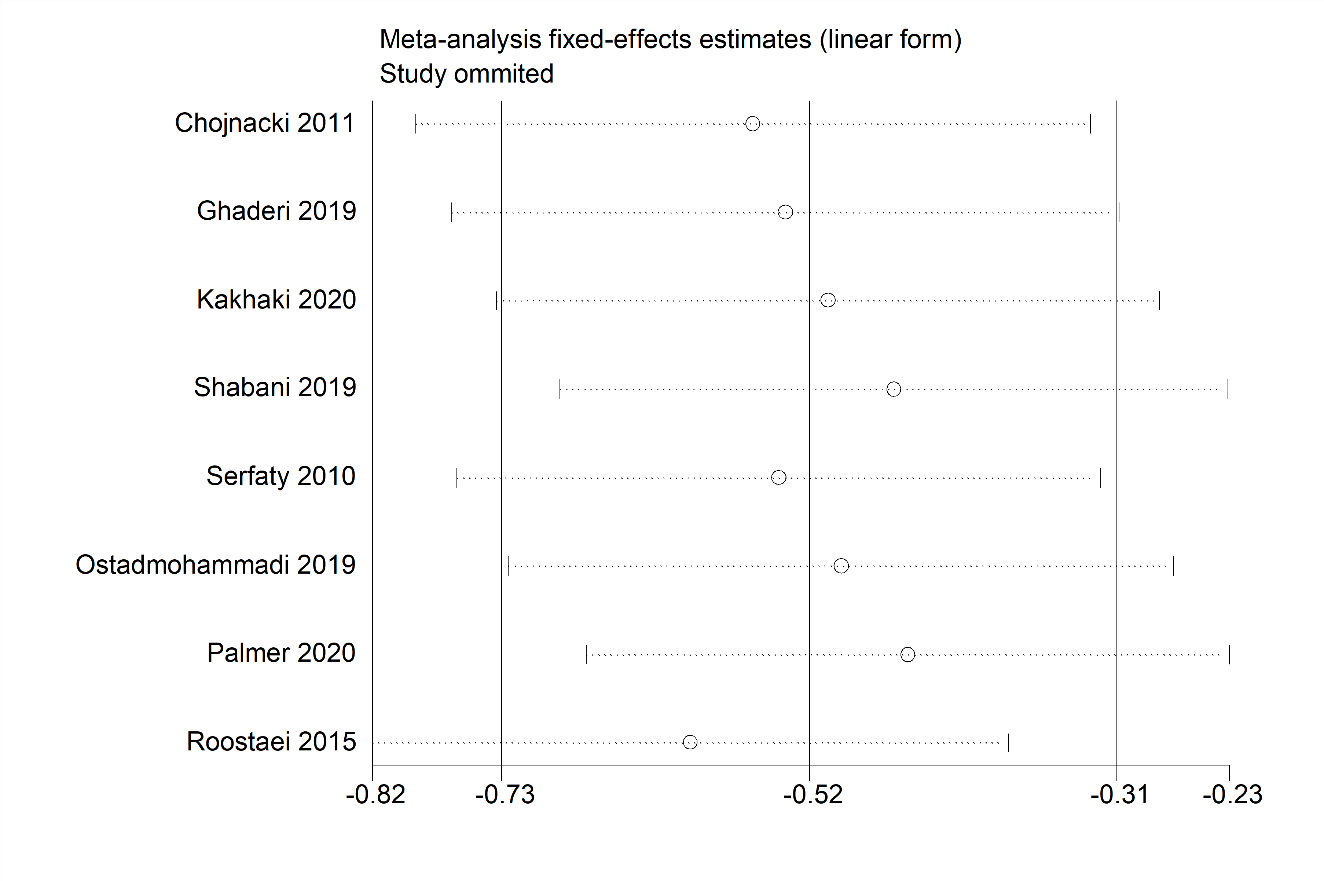


**Figure S12** Sensitivity analysis on BDI score


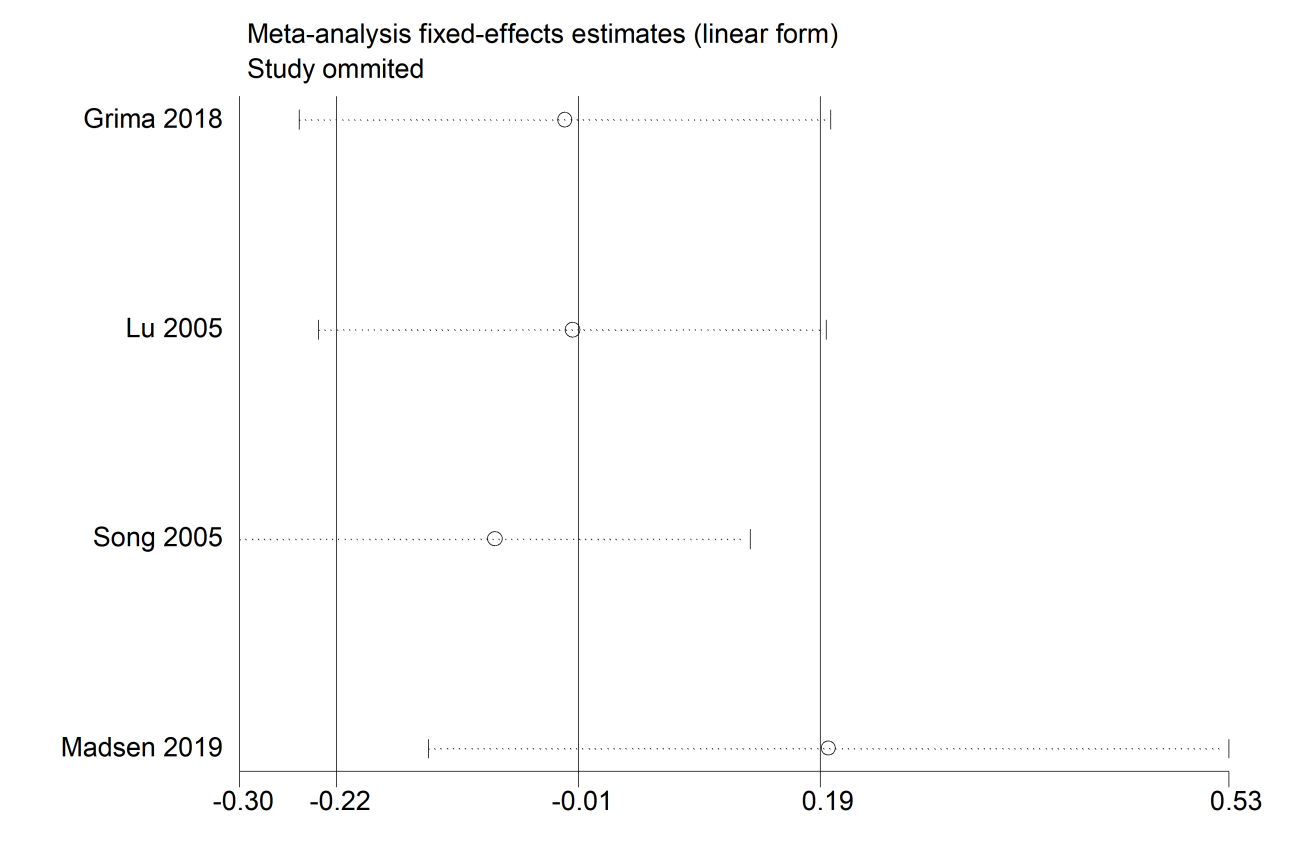


**Figure S13** Sensitivity analysis on HADS-D score


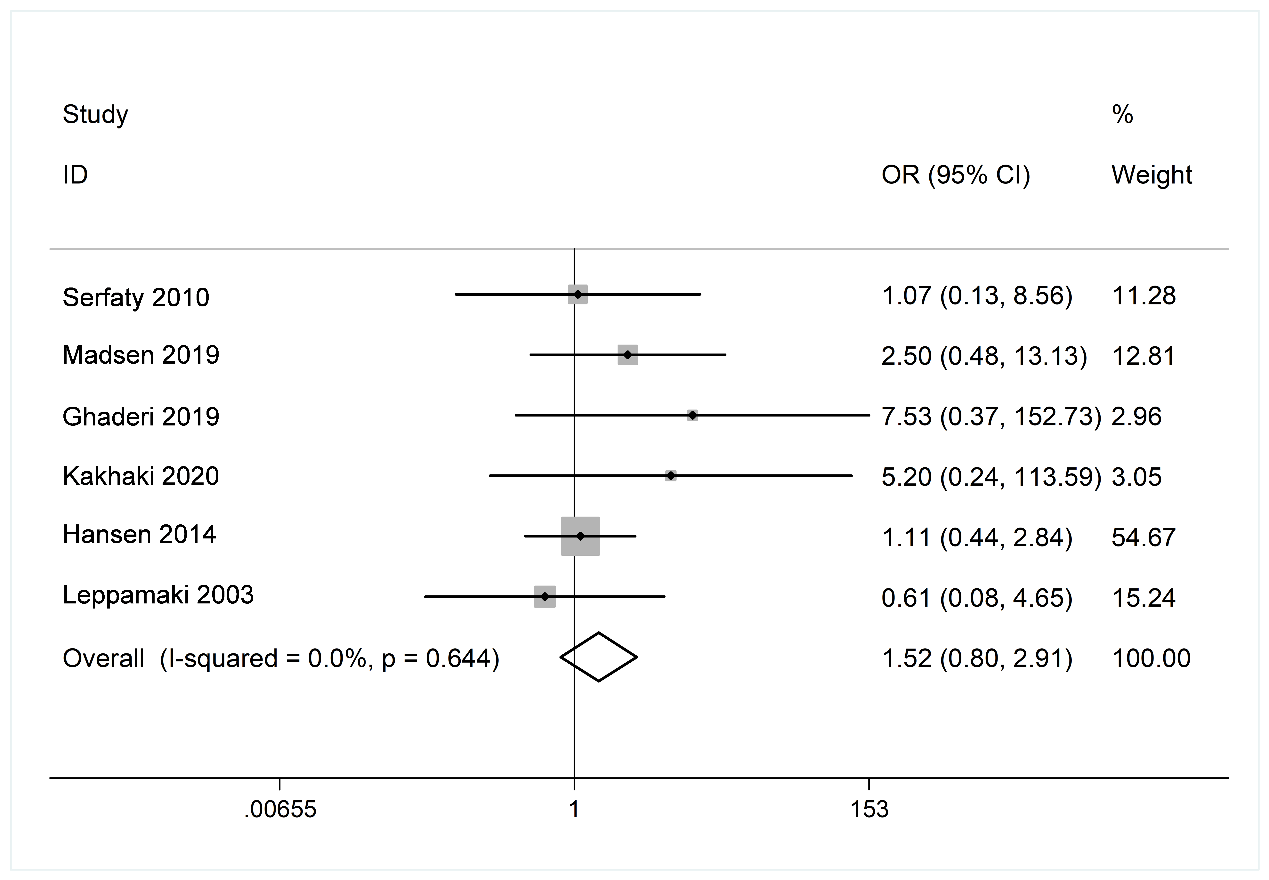


**Figure S14** Adverse event analysis. OR, Odds Ratio; CI, confidence interval
